# Rainbow Trout (*Oncorhynchus Mykiss*) Intestinal Epithelial Cells as a Model for Studying Gut Immune Function and Effects of Functional Feed Ingredients

**DOI:** 10.3389/fimmu.2019.00152

**Published:** 2019-02-06

**Authors:** Jie Wang, Peng Lei, Amr Ahmed Abdelrahim Gamil, Leidy Lagos, Yang Yue, Kristin Schirmer, Liv Torunn Mydland, Margareth Øverland, Åshild Krogdahl, Trond M. Kortner

**Affiliations:** ^1^Department of Basic Sciences and Aquatic Medicine, Faculty of Veterinary Medicine, Norwegian University of Life Sciences (NMBU), Oslo, Norway; ^2^Department of Animal and Aquacultural Sciences, Faculty of Biosciences, Norwegian University of Life Sciences (NMBU), Oslo, Norway; ^3^Eawag, Swiss Federal Institute of Aquatic Science and Technology, Dübendorf, Switzerland; ^4^EPF Lausanne, School of Architecture, Civil and Environmental Engineering, Lausanne, Switzerland; ^5^ETH Zürich, Institute of Biogeochemistry and Pollutant Dynamics, Zurich, Switzerland

**Keywords:** RTgutGC, *in vitro* model, lipopolysaccharide, functional feed ingredients, mucosal immune responses, gut barrier

## Abstract

The objective of this study was to evaluate the suitability of the rainbow trout intestinal epithelial cell line (RTgutGC) as an *in vitro* model for studies of gut immune function and effects of functional feed ingredients. Effects of lipopolysaccharide (LPS) and three functional feed ingredients [nucleotides, mannanoligosaccharides (MOS), and beta-glucans] were evaluated in RTgutGC cells grown on conventional culture plates and transwell membranes. Permeation of fluorescently-labeled albumin, transepithelial electrical resistance (TEER), and tight junction protein expression confirmed the barrier function of the cells. Brush border membrane enzyme activities [leucine aminopeptidase (LAP) and maltase] were detected in the RTgutGC cells but activity levels were not modulated by any of the exposures. Immune related genes were expressed at comparable relative basal levels as these in rainbow trout distal intestine. LPS produced markedly elevated gene expression levels of the pro-inflammatory cytokines *il1b, il6, il8*, and *tnfa* but had no effect on ROS production. Immunostaining demonstrated increased F-actin contents after LPS exposure. Among the functional feed ingredients, MOS seemed to be the most potent modulator of RTgutGC immune and barrier function. MOS significantly increased albumin permeation and *il1b, il6, il8, tnfa*, and *tgfb* expression, but suppressed ROS production, cell proliferation and *myd88* expression. Induced levels of *il1b* and *il8* were also observed after treatment with nucleotides and beta-glucans. For barrier function related genes, all treatments up-regulated the expression of *cldn3* and suppressed *cdh1* levels. Beta-glucans increased TEER levels and F-actin content. Collectively, the present study has provided new information on how functional ingredients commonly applied in aquafeeds can affect intestinal epithelial function in fish. Our findings suggest that RTgutGC cells possess characteristic features of functional intestinal epithelial cells indicating a potential for use as an efficient *in vitro* model to evaluate effects of bioactive feed ingredients on gut immune and barrier functions and their underlying cellular mechanisms.

## Introduction

The fish intestine is a multifunctional organ responsible for key physiological processes such as digestion, absorption of nutrients, and osmoregulation (1). Furthermore, the intestine has an important immunological role and constitutes a physical barrier against pathogens (1). In order to secure optimal gut health and function in farmed fish, there is now particular focus on various feed additives including functional feed ingredients that are branded not only in terms of their nutritional value, but also based on their health promoting and disease preventing properties. These functional feed ingredients could include intact microbes (e.g., probiotic organisms), mixed or purified extracts from microbial or plant structural components [e.g., mannanoligosaccarides (MOS), beta-glucans], metabolites (e.g., nucleotides) or even conventional nutrients, if their dietary inclusion is higher than the animal's requirement. Functional feeds are typically applied during predicted stressful events or challenging farming conditions, such as grading, sea water transfer, vaccination, and during critical life stages to help the animal ward off pathogens and secure good health (2). Functional feed ingredients are generally believed to exert their main actions locally within the gut, and may have direct modulatory effects on gut microbiota (3), gut barrier, immune, and/or metabolic functions (4–7). For example, nucleotides are of crucial importance for a whole range of normal intestinal functions, such as growth, nutrient metabolism, immune system, tissue repair, and development (8). Beta-glucans can increase cellular and humoral immune responses in immune cells and epithelial tissues of fish (9–11). MOS as an immune modulator has a close relation to pathogen colonization blocking and immune system regulation, as well as improving intestinal morphology and the epithelial brush border (10, 12, 13).

Current knowledge regarding mechanisms underlying effects of functional feed ingredients on fish gut health and function is, however, limited largely due to a lack of targeted research tools. The use of *in vitro* approaches, such as appropriate cell lines, would facilitate further research on basic functions of the digestive tract and effects of functional feed ingredients on host intestinal immune, barrier and digestive function. It would also reduce the current dependence on large-scale feeding trials, thus contributing to a shift toward 3R studies within fish nutrition research. In mammalian research, intestinal cell lines have proven to be valuable tools for exploration of basic mechanisms of gut function and health and interactions with dietary components. For example, nucleotide supplements in human Caco-2 and rat IEC-6 cell lines have been observed to strengthen intestinal maturation and growth (14). Beta-glucans and plant flavonoids can suppress nuclear factor-kB transactivation, regulate immune response, and strengthen intestinal epithelial barrier function in human Caco-2 cells (15–17).

Until recently, no relevant intestinal cell lines from fish have been available, but promising cultures have been established based on the rainbow trout (*Oncorhynchus mykiss*) intestinal derived cell line RTgutGC (18). Since their initial isolation, RTgutGC cells have been relatively well-characterized and are now routinely grown as monolayers on permeable supports, leading to a two-compartment intestinal barrier model consisting of a polarized epithelium. The system is divided into an upper (apical) and a lower (basolateral) compartment, thereby mimicking the intestinal lumen and the portal blood, respectively. Reported structural and functional features of the RTgutGC cells include tight junction and desmosome formation between adjacent cells, development of transepithelial resistance and polarization over time to exhibit epithelial and brush border characteristics (18–20). The cell line has, as such, been proposed as a physiologically adequate fish intestinal epithelial model, equivalent to the Caco-2 cell line for human intestinal epithelium (20, 21), and has been used recently in studies on fish intestinal immune and barrier function (18, 22, 23).

The objective of this study was to evaluate the suitability of the RTgutGC cells as an *in vitro* model for studies of gut immune function and effects of functional feed ingredients. Effects of a prototype pathogen-associated molecular pattern (PAMP), lipopolysaccharide (LPS), and three functional ingredients commonly applied in commercial fish feeds (nucleotides, MOS, and beta-glucans) were evaluated by diverse analyses, including cell viability measurements and proliferation, brush border digestive enzyme activity, barrier function, ROS production, morphology, and relevant gene and protein expression.

## Materials and Methods

### RTgutGC Cell Culture

Routine RTgutGC cell cultivation was based on the description by Kawano et al. (18). Briefly, RTgutGC cells were cultured in 75-cm^2^ flasks (TPP, Trasadingen, Switzerland). L-15 complete medium (L-15/C), i.e., Leibovitz's L-15 medium without Phenolred (21083, Gibco Invitrogen, Basel, Switzerland) supplemented with 10% bovine serum (F7524, Sigma Aldrich, Buchs, Switzerland) and gentamicin (15710-049, Invitrogen, Basel, Switzerland) with a final concentration of 100 μg/mL, was used to culture cells in a 20°C incubator under normal atmosphere. Cells were split in a 1:2 ratio using trypsin (0.25% in PBS w/o Ca^2+^, Mg^2+^; L0910; Biowest; Nuaillé, France) after reaching confluency.

For cells grown on conventional culture plates without inserts, 1 mL or 3.5 mL cell suspensions (1.5 × 10^5^ cells/mL, 78,947 cells per cm^2^ for 24-well plates and 54,688 cells per cm^2^ for 6-well plates) were seeded in 24-well (No.662160, Greiner-Bio-one, Frickenhausen, Germany) or 6-well plates (No. 657960, Greiner-Bio-one, Frickenhausen, Germany), respectively, and were cultured to reach at least 80 % confluency before use (3–4 days).

For the two-compartment intestinal barrier model, RTgutGC cells were cultured as described previously (19, 20). Briefly, 24-well plates with 33.6 mm^2^ transwell inserts (No. 662 630, Greiner-Bio-one, Frickenhausen, Germany) and 6-well plates with 425.4 mm^2^ transwell inserts (No. 657 630, Greiner-Bio-one, Frickenhausen, Germany) with pore sizes of 3 μm were used to simulate gut lumen (apical /upper chamber) and portal blood (basolateral /lower chamber). Cells were seeded adding 300 μL or 3.5 mL cell suspension (8 × 10^4^ cells/mL, 71,429 cells per cm^2^ for 24-well plates and 65,820 cells per cm^2^ for 6-well plates) in the apical chamber of 24-well or 6-well plates, respectively. Then, 1 or 3.5 mL of L-15/C were added into the basolateral chamber of 24-well or 6-well plates, respectively. The apical and basolateral medium was changed once per week for a total of 28 days.

### Exposure Design

Stock solutions were prepared for LPS and the functional ingredients. LPS (L2630, Sigma, Norway) stock solution was prepared to 1 mg/mL in L15/ex medium. The L15/ex medium contains only the inorganic salts, galactose, and pyruvate concentrations of L-15 (24). Nucleotides (T25-1KT, Sigma, Norway) stock solution was prepared to 10 mg/mL using milliQ water. MOS (Active MOS extracted from yeast, Biorigin, São Paulo, Brazil) stock solution was prepared to 20 mg/mL using sterile PBS, and then sonicated in a water bath (30 s/3 times) and centrifuged (500 × g/ 5 min). The supernatant was subsequently transferred into new vials and stored at −20°C according to previous descriptions (13). Beta-glucans (G5011, Sigma, Norway) stock solution was prepared to 2 mg/mL in sterile PBS according to previous reports (23).

For all exposure tests performed with the two-compartment intestinal barrier model, the stock solutions of LPS and the functional ingredients were diluted in mucosal saline, prepared according to Genz et al. (25) (Supplementary Table 1), and added to the apical chamber in order to mimic intestinal lumen conditions. Before performing the exposure tests with LPS and the functional ingredients, L-15/C and mucosal saline acted as exposure medium for RTgutGC cells to evaluate whether the mucosal saline affected cell viability. For exposure tests performed with conventional plates, working solutions were prepared by diluting in mucosal saline or L15 medium, depending on the analytical assay as specified below. To select final working concentrations for further analysis, LPS and the functional ingredients were tested at a range of different concentrations in 6 h exposures in 24-well-conventional plates without inserts (Supplementary Table 2).

### Assessment of Cell Viability

Alamar Blue (AB, DAL1025, Invitrogen, Basel, Switzerland) and 5-carboxyfluorescein diacetate acetoxymethyl ester (CFDA-AM, C1345, Invitrogen, Basel, Switzerland) were used to measure cell viability (24, 26). AB was used to measure cell metabolic activity, whereas CFDA-AM was used to measure cell membrane integrity. After 6 h of incubation, stimulant working solutions were discarded, cells were washed twice using 1 mL PBS and subsequently, a volume of 400 μL of fresh AB and CFDA-AM were added to each well. The plates were then incubated at 20°C for 30 min in the dark before measurement. The Cytation 3 plate reader (Bio Tek Instruments, Winooski, USA) was used to measure the fluorescence of AB (λex = 530 nm; λex = 595 nm) and CFDA-AM (λex = 493 nm; λex = 541 nm).

### Measurement of Transepithelial Electrical Resistance (TEER)

As a quality measure of monolayer formation, TEER was measured in RTgutGC cells grown in 24-well-culture plates with membrane inserts at day 1, 7, 14, and 28. Additionally, TEER was measured in RTgutGC cells exposed to LPS and the functional ingredients for 6 h after 28 days of culture on transwell membrane inserts in 6-well plates. TEER levels were measured using an EVOM Voltohmmeter with STX2 electrode and Endohm-6 electrode (World Precision Instruments, Berlin, Germany) as described by Geppert et al. (19). TEER was calculated by subtracting the values without cells from the values with cells. TEER values were given as Ω × cm^2^.

### Brush Border Membrane Enzyme Activity

After 3–4 days of culture on conventional 24-well plates, RTgutGC cells were exposed to LPS and the functional ingredients for 6 h. After discarding the mucosal saline with LPS or functional ingredients, cells were harvested by trypsination and centrifugation. Cell pellets were reconstituted in 1 mL ice-cold 2 mM Tris/50 mM mannitol pH 7.1, containing phenyl-methyl-sulphonyl fluoride (P-7626, Sigma, Norway) as serine protease inhibitor. Brush border membrane enzyme activities, i.e., leucine amino peptidase (LAP) and maltase, were subsequently measured. LAP activity was analyzed colorimetrically with a commercial kit (NO. 251, Sigma, Norway) using L-leucine-β-naphthylamide as substrate according to the methods described by Krogdahl et al. (27). Maltase activity was measured using maltose as substrate according to the description of Dahlquist (28). Total protein concentrations were determined using a Bio-Rad Protein Assay (Bio-Rad Laboratories, Munich, Germany). Enzyme activities were expressed as mol substrate hydrolysed per hour per mg protein.

### Albumin Translocation Assay

After 28 days of culture on transwell-membrane inserts in 6-well plates, RTgutGC cells with an initial seeding density of 8 × 10^4^ cells/mL (65,820 cells per cm^2^) were exposed to LPS and the functional ingredients for 6 h. The permeation of fluorescent-labeled albumin was then used to evaluate the barrier potential of the cells. 20 μL albumin (Alexa FluorTM 488 Bovine Serum Albumin, Thermo Fisher Scientific, USA) was added into the apical chamber of each well, and 250 μL of culture medium was collected from the basolateral chamber at the following intervals: 10, 30, 45, 60, and 90 min and temporary stored in the dark at 20 °C. After collecting all the samples, 100 μL of each sample was added to a 96- well black plate (M5061-40EA, Sigma, Norway) in duplicate, and fluorescence was measured using a Cytation 3 plate reader (Bio Tek Instruments, Winooski, USA) equipped with a 490 excitation and 525 emission filter.

### Quantitative Real Time PCR (qPCR)

After 28 days of culture on transwell membrane inserts in 6-well plates, RTgutGC cells were exposed to LPS and the functional ingredients for 6 h, and subsequently harvested for gene expression profiling. After discarding the mucosal saline with LPS or functional ingredients, 1 mL of TRIzol (Invitrogen, Thermo Fisher Scientific, USA) was added to each apical chamber. Cells were collected by scarping and flushing the membrane inserts 10 times with the TRIzol solution. The cell homogenate was transferred into a 1.5 mL Eppendorf tube, snap frozen in liquid N_2_ and subsequently stored at −80 °C until RNA extraction. Gene expression levels in RTgutGC cells were compared with those of rainbow trout tissues by using total RNA samples from liver, pyloric, mid and distal intestine obtained from a fresh-water stage female rainbow trout as previously described (29). RNA was subsequently purified using a PureLink RNA mini Kit (Invitrogen, Thermo Fisher Scientific, USA). RNA purity and concentration were measured using an Epoch Microplate Spectrophotometer (BioTeK Instruments, Winooski, USA). The RNA integrity was verified using a 2100 Bioanalyzer in combination with RNA Nano Chip (Agilent Technologies, Santa Clara, USA). First-strand complementary DNA was synthesized from 1.0 μg total RNA from all samples using SuperScript IV VILO Master Mix (Invitrogen™, ThermoFisher Scientific). Negative controls were performed in parallel by omitting RNA or enzyme.

Twelve target genes with important functions related to immunity, barrier function and metabolism were profiled. The qPCR primers were designed using Primer3web software version 4.0.0 (http://primer3.ut.ee/) or obtained from the literature. Primer details are shown in Supplementary Table 3. All primer pairs were first used in gradient reactions in order to determine optimal annealing temperatures. To confirm amplification specificity, the PCR products from each primer pair were subjected to melting curve analysis and visual inspection of the PCR products by agarose gel electrophoresis. PCR efficiency for each gene assay was determined using 2-fold serial dilutions of randomly pooled complementary DNA. The expressions of individual gene targets were analyzed using the LightCycler 96 (Roche Diagnostics, Basel, Switzerland). Each 10 μl DNA amplification reaction contained 2 μl PCR grade water, 2 μl of 1:10 diluted complementary DNA template, 5 μl LightCycler 480 SYBR Green I Master (Roche Diagnostics) and 0.5 μl (10 mM) of each forward and reverse primer. Each sample was assayed in duplicate, including a no-template control. The three-step qPCR run included an enzyme activation step at 95°C (5 min), forty to forty-five cycles at 95°C (10 s), 60°C (10 s), and 72°C (15 s) and a melting curve step. Target gene expression was normalized to the geometric average of beta-actin (*actb*) and ribosomal protein s20 (*rps20*) after confirming reference gene intra- and interspecific stability (30). Mean normalized expression of the target genes was calculated from raw Cq values by relative quantification (31).

### Cell Proliferation Assay

The ability of RTgutGC cells to close a gap during exposure to LPS, MOS and beta-glucans was investigated in a cell proliferation assay by using 2-well-culture inserts (80241, Ibidi GmbH, Martinsried, Germany). The inserts were placed on a conventional cell culture surface, i.e., a μ-Dish 35 mm (81156, Ibidi GmbH, Martinsried, Germany) creating two wells, which were separated by a rubber partition. Approximately 10,000 cells in 70 μL L-15/C were seeded into each well. The cultures were incubated at 20 °C for 2 days until confluence. Then, the rubber partition was removed to create a 500 μm gap between the cells. Immediately, LPS (50 μg/mL), MOS (4 mg/mL), beta-glucans (100 μg/mL) and PBS (control), all dissolved in L-15 medium, were added to the cultures and phase contrast pictures were captured at day 0, 1, 2, and 4 (or until the gap was closed) using a ZEISS Axio microscope (with Axiocam 105 color). The image were processed using ImageJ (32). In brief, all images were first adjusted using Adjust tool for achieving a clear contrast between the cell-free area and area covered by cells. Subsequently, the cell-free area was measured using Analyze particles tool. The cell proliferation rate was calculated by dividing the cell-free area at each time point with the cell-free area at day 0.

### Oxidative Stress Detection and Substrate Uptake Assay

After 3–4 days of culture on conventional 6-well plates, RTgutGC cells were exposed to LPS and the functional ingredients for 6 h. After discarding the mucosal saline with LPS or functional ingredients, cells were harvested by trypsination and centrifugation. Cell pellets were reconstituted in 5% FBS in PBS for ROS generation measurement. CellROX® (C10444, Thermo Fisher, Waltham, USA) reagent was added to the cell suspensions at a final concentration of 5 mM, followed by incubation at room temperature for 30 min. After the incubation, cells were washed three times with ice-cold phosphate buffered saline (PBS) and ROS generation was analyzed by flow cytometry (Beckman Coulter Gallios). At least 10,000 events were collected for each sample. Data were analyzed using Kaluza software v.2.1 (Beckman Coulter) and gated using Side scatter (SSC) (granularity) and Forward scatter (FSC) (size) parameters. Discrimination of aggregates from single cells was performed using side scatter-W (SSC-W) vs. side scatter (SSC). ROS was measured at 650/675 nm (FL3).

Fluorescence conjugated Zymosan (Z23373, Thermo Fisher Scientific, USA) and albumin (Alexa FluorTM 488 Bovine Serum Albumin, Thermo Fisher Scientific, USA) were added into culture media at 20 and 12.5 μg/ml, respectively for cells growing in 6-well-conventional plates. Cells were trypsinized and centrifuged after 1, 1.5, and 3 h after adding substrates, respectively. Cell pellets were reconstituted in 5% FBS in PBS before flow cytometry (Beckman Coulter Gallios) was performed to analyze the cells with or without fluorescence at 495/519 nm.

### Immunocytochemistry of F-actin Content

For morphological characterization, confocal laser microscopy was used for imaging. RTgutGC cells in L-15/C were seeded in an 8 chamber tissue cultured treated glass Falcon CultureSlide® (Corning, New York, USA) at a density of 150,000 cells per chamber. When reaching 80% confluence, cells were washed with Dulbecco's Phosphate-Buffered Saline (DPBS) and treated with LPS (50 μg/mL), MOS (4 mg/mL), beta-glucans (100 μg/mL), and PBS (control), all dissolved in L-15 medium. After 6 h, cells were washed with DPBS and fixed with 3% paraformaldehyde (Sigma-Aldrich) for 20 min at 4°C. Following fixation, the cells were permeabilized with 0.1% Triton X-100 (Sigma-Aldrich, St. Louis, USA) for 10 min at room temperature. Cells were then incubated in blocking buffer (BB) (10% goat serum, 3% bovine serum albumin, and 0.1% Triton X-100 in DPBS) for 1 h at room temperature. Afterwards, cells were incubated with phalloidin (R425, Thermo Fisher) for F-acin staining according to the manufacturer's instruction. After staining, cells were washed three times for 3 min with DBPS and left to air dry. Once dry, plastic chambers were removed from the slides. Three drops of mounting medium, Fluoroshield (Sigma-Aldrich), containing DAPI were added to the slides, followed by covering with coverslip. The image was analyzed by ImageJ software to investigate the morphology change of the cells under different treatments. Three random pictures were taken from cells under respective treatments. Individual cell numbers were counted based on DAPI-stained nuclear numbers manually. F-actin contents were subsequently calculated by the total fluorescence intensity of phalloidin divided by number of the cells.

### Protein Expression of E-cadherin, Aquaporin 8, and Hsp70 by Western Blot Analysis

RTgutGC cells were seeded on 6-well plates and grown until confluence before 6 h exposure to LPS (50 μg/mL), MOS (4 mg/mL), beta-glucans (100 μg/mL), and PBS (control), all dissolved in L-15 medium. Cells were harvested by trypsinization and centrifugation and protein was extracted using PARIS Kit (AM 1921, Thermo Fisher) according to the manual. Protein concentrations were measured using Bradford protein assay kit (Bio-Rad, Hercules, California, United States) and 10 or 20 μg of the protein were loaded on SDS page gels. After 40 min electrophoresis at 100 voltage, proteins were transferred to PVDF membranes, blocked with 5% dry milk for 1 h at room temperature, and incubated consecutively with E-cadherin monoclonal antibody (#701134, Thermo Fisher), Heat shock protein 70 (Hsp70) monoclonal antibody (MA3-008, Thermo Fisher), or Aquaporin 8 (Aqp8) polyclonal antibody (kindly provided by Prof. Steffen S. Madsen, Institute of Biology, University of Southern Denmark). After 3 times washing in PBS and incubation of HRP conjugated secondary antibody, the signal was visualized with Bio-Rad Gel Doc system after adding ECL detection reagents (GERPN2209, Sigma-Aldrich) to the membrane. Due to the potential influence of treatments on candidate reference protein expression, the total membrane protein content was visualized with Ponceau S (P3504, Sigma) and used as a qualitative loading control.

### Statistical Analysis

All data were tested for normality and variance homogeneity using histogram and “residual by predicted” plot, respectively, using JMP Pro 13.0.0 (SAS Institute, United States). When necessary, the data were transformed to achieve normal distribution. Further statistical analyses and graphics were made using GraphPad Prism 7 (GraphPad Software, La Jolla, California, United States). The flow cytometry figures were made by Kaluza (Beckman Coulter). Data of albumin translocation and cell proliferation rate were analyzed using two-way ANOVA using time and treatment as class variables followed by Dunnett multiple comparisons tests. Other data were analyzed using one-way ANOVA followed by Dunnett multiple comparisons tests. Data were calculated as mean ± SEM of two or three independent experiments with 3 or 4 technical well or insert replicates (depending on analytical assays, see specifications in figure legends). Asterisks denote the level of statistical significance (^*^P < 0.05, ^**^P < 0.01, ^***^ *P* < 0.001).

## Results

### Key Features of RTgutGC Cells Exposed in Mucosal Saline in Conventional Culture Plates and Transwell Membranes

**Figure 1:**
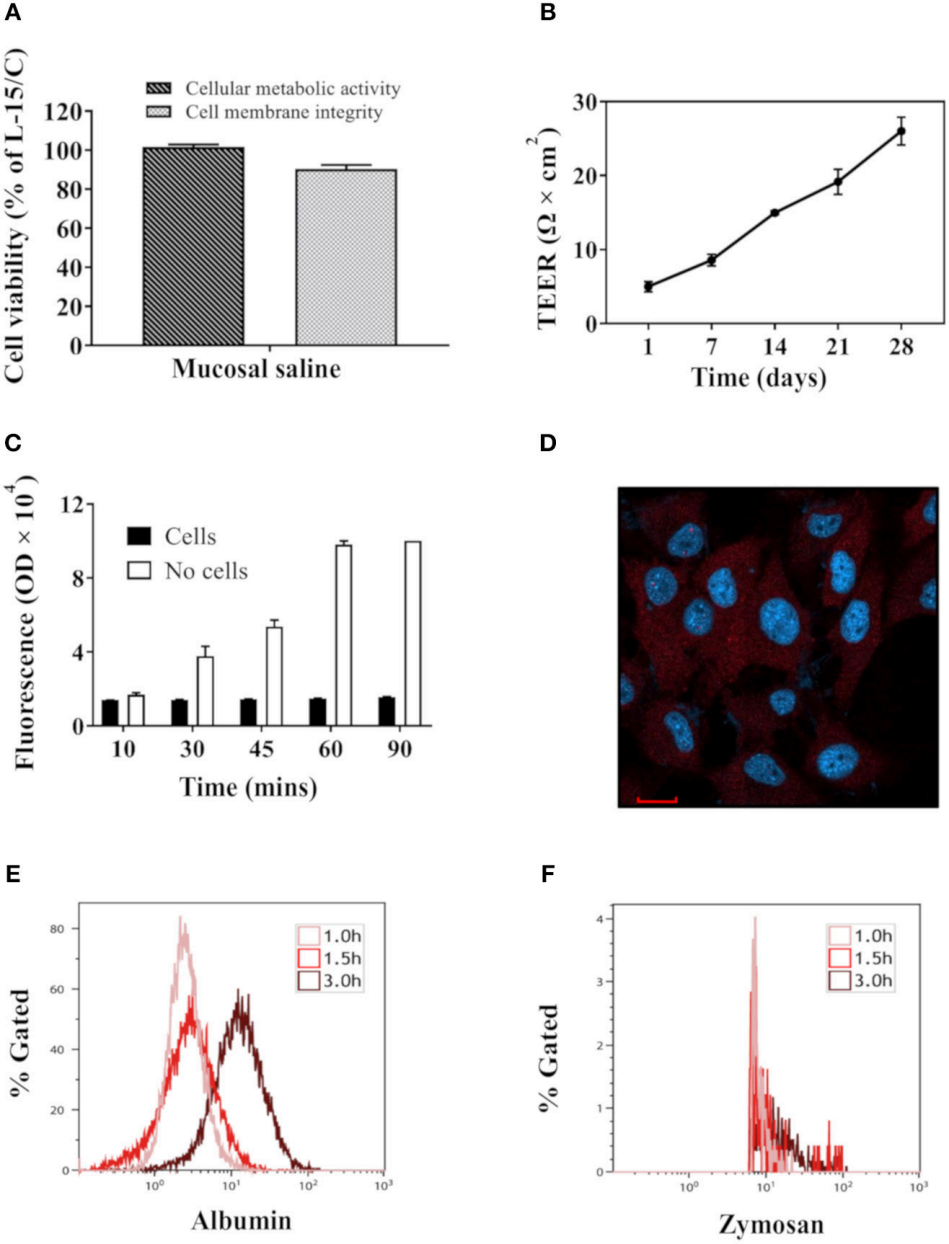
Key features of RTgutGC cells grown on conventional culture plates or transwell membranes. **(A)** Viability of RTgutGC cells in 24-well-culture plates with 1.5 × 10^5^ cells/mL (78,947 cells per cm^2^) exposed to mucosal saline for 12 h. **(B)** TEER of RTgutGC cells grown up to 4 weeks in 24-well-culture plates with membrane inserts at initial density of 8 × 10^4^ cells/mL (71,429 cells per cm^2^). **(C)** Fluorescent levels in basolateral media after fluorescent albumin exposure into apical chamber in 24-well-transwell membrane plates with or without RTgutGC cells. **(D)** Confocal fluorescence microscopy images of the tight junction protein claudin 3 (red) and the nuclei (blue) in RTgutGC cells grown on conventional culture plates. **(E,F)** Uptake of albumin **(E)** and zymosan **(F)** during the 3 h exposure time with cells cultured in conventional 6-well plates. For both panels **(E,F)**, X axis shows the fluorescence signal from albumin or zymosan in cells. Y axis shows the percentage of albumin/zymosan positive cells out of total live cell population. Data represent mean ± SEM of two independent experiments with 3–4 technical replicates each (wells or inserts). Scale bar = 100 μm.

Compared with RTgutGC cells cultivated in L-15/C medium, cells cultivated in mucosal saline maintained above 80% cell viability after 12 h exposure (Figure 1A). When RTgutGC cells were grown on transwell membrane inserts in 24-well plates, TEER levels increased steadily and reached about 26 Ω × cm^2^ after 4 weeks of culture (Figure 1B). After addition of fluorescent albumin to mucosal saline solution in the apical chamber, basolateral fluorescence levels increased steadily with time when no cells were seeded on the membrane whereas with cells, low fluorescence was observed over the 90 min observation period, demonstrating that the RTgutGC cells formed a barrier and strongly attenuated albumin translocation from the apical to the basolateral chamber (Figure 1C). Confocal fluorescence microscopy images of RTgutGC cells grown on conventional culture plates illustrated presence of the tight junction protein Claudin 3 (red) and the nuclei (blue) (Figure 1D).We also investigated the uptake of albumin and zymozan in RTgutGC cells, grown on conventional support, as a character of functional enterocytes. During a time course of 3 h, albumin uptake into RTgutGC cells increased (Figure 1E). However, RTgutGC cells did not take up zymosan as shown in Figure 1F.

### Effects of LPS and Functional Ingredients

#### Cell Viability

**Figure 2:**
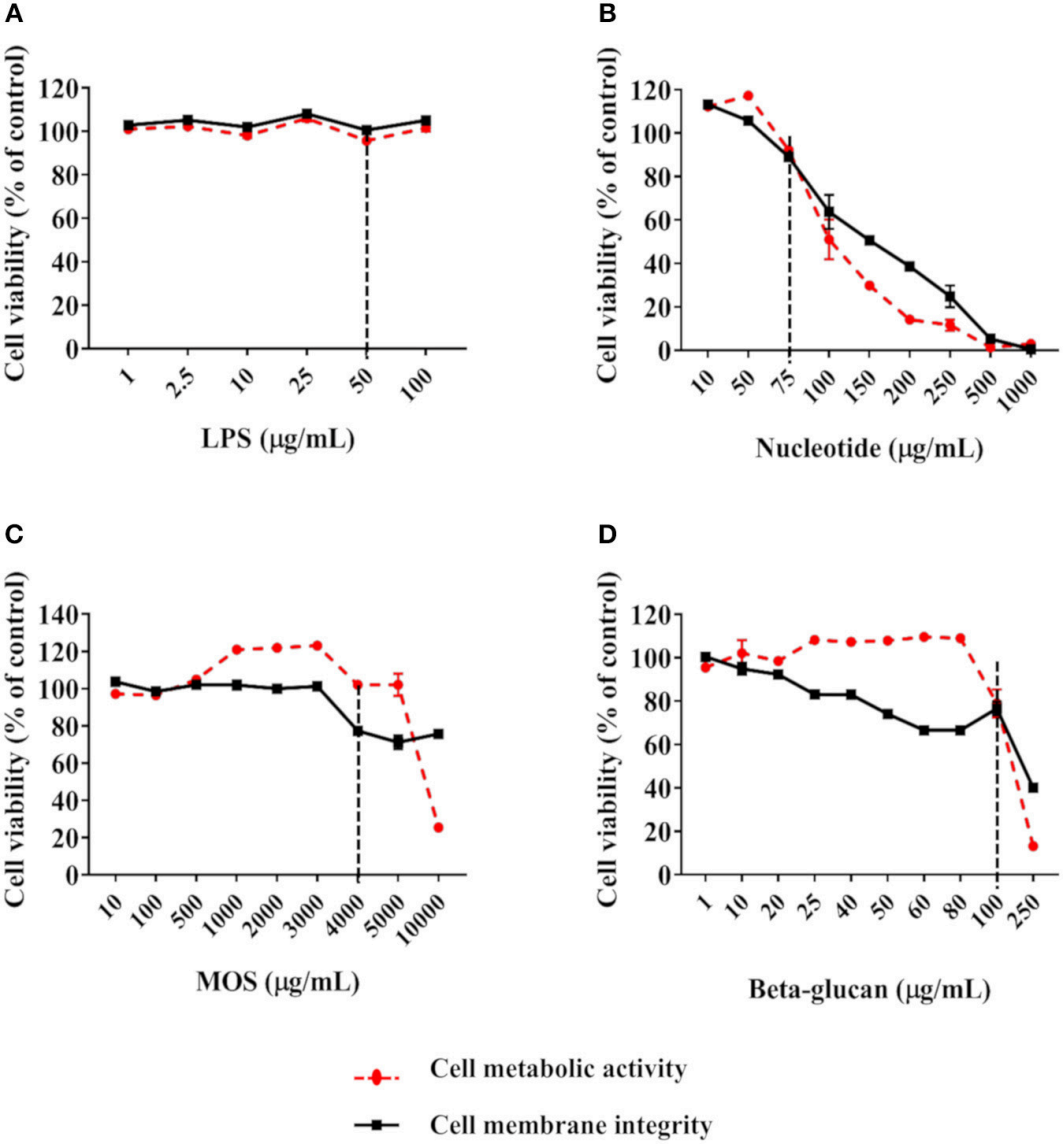
Viability of RTgutGC cells exposed to LPS **(A)**, nucleotides **(B)**, MOS **(C)**, and beta-glucan **(D)**. Breaking lines indicate cut-off level of about 80% cell viability compared to control cells. For LPS, data represent mean ± SEM of two independent experiments with 3 technical well-replicates. For nucleotides, MOS and beta-glucan, two independent experiments with 3 technical well-replicates each and concentrations as shown in Supplementary Table 1 were conducted. For concentrations evaluated in both experiments, data represent mean ± SEM of the two experiments. For concentrations only evaluated in one of the experiments, single mean values are plotted.

Using a cell viability cut-off level of 80% compared to control cells, 6 h of exposure to 50 μg/mL LPS (Figure 2A), 75 μg/mL nucleotides (Figure 2B), 4 mg/mL MOS (Figure 2C) and 100 μg/mL beta-glucans (Figure 2D) were selected as final working concentration for further analysis.

#### TEER and Albumin Translocation

**Figure 3:**
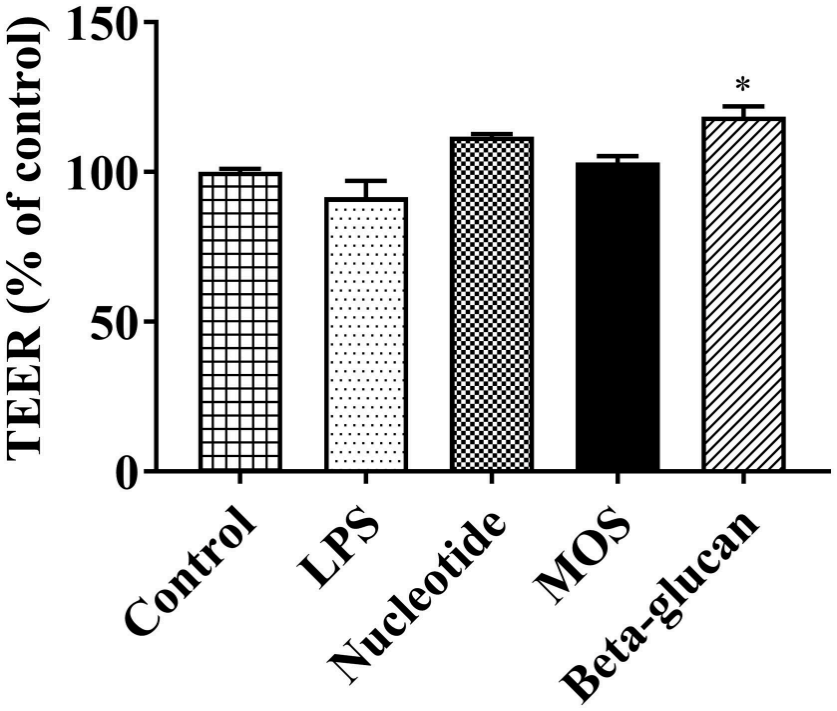
TEER levels of RTgutGC cultures exposed to LPS and functional ingredients in 6-well-transwell membrane inserts for 6 h. Data are expressed as percent of control cells and represent mean + SEM of two independent experiments with 3 technical insert replicates each. Asterisks denote treatment groups statistically different to the control (^*^P < 0.05).

After 6 h of exposure to beta-glucans, TEER levels increased significantly compared to control (Figure 3, *P* **<** 0.05). Other treatments had no effect on TEER levels (Figure 3, original TEER values seen in Supplemental Figure 1).

**Figure 4:**
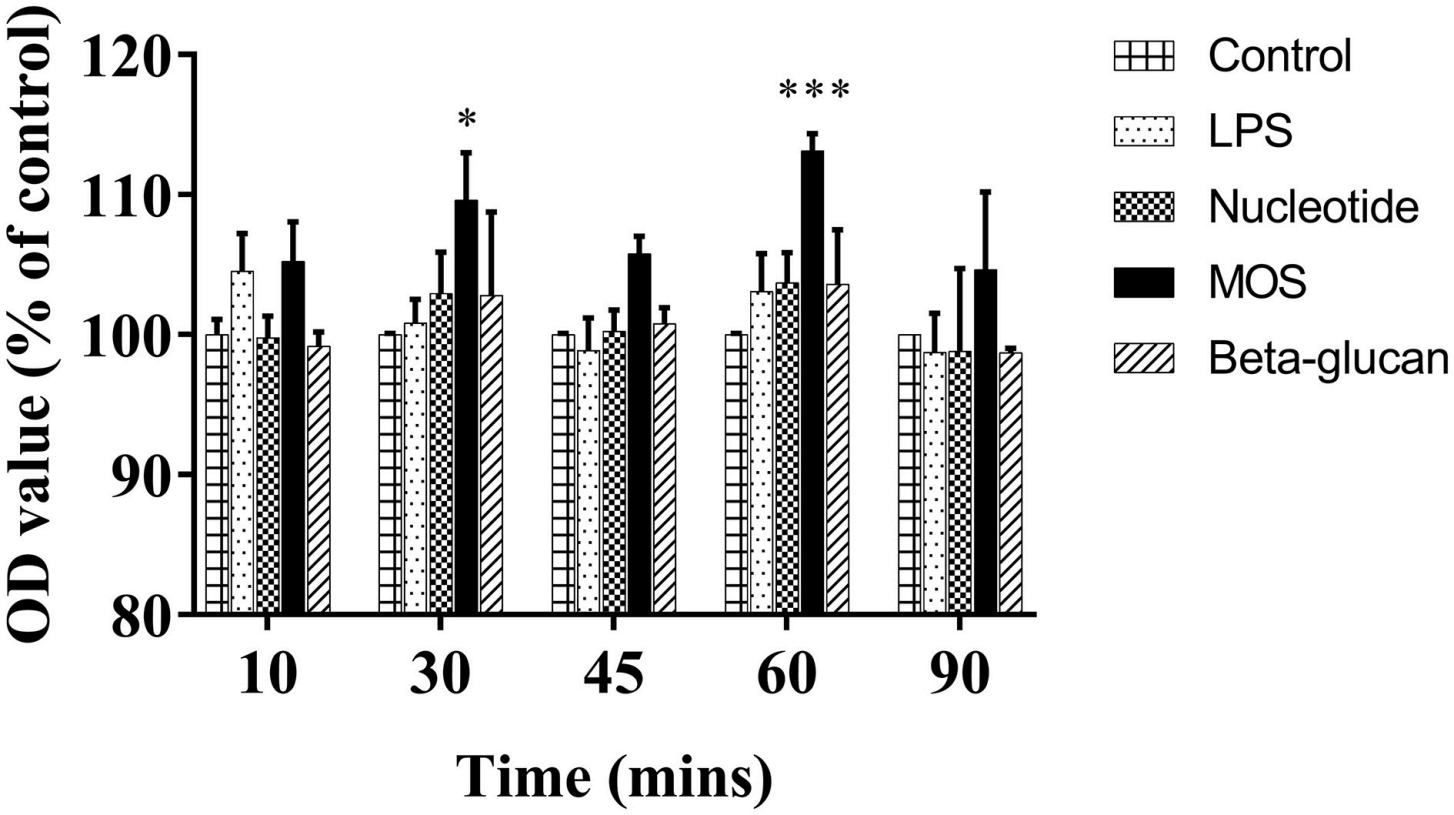
Fluorescent levels in basolateral media after fluorescent albumin exposure into apical chamber in 24-well transwell membrane with RTgutGC cells exposed to LPS and functional ingredients for 6 h. Data are expressed as percent of control cells and represent mean + SEM of two independent experiments with 3 technical insert replicates each. Asterisks denote treatment groups statistically different to the control at the same time point (^*^P < 0.05, ^***^P < 0.001).

After 6 h of exposure to MOS increases in basolateral albumin fluorescent levels were observed compared to control cells, significantly at 30 and 60 min time points (*P* < 0.05). No significant effects on the fluorescent level were observed for other treatments (Figure 4, *P* **>** 0.05).

#### Brush Border Membrane Enzymatic Activity

**Figure 5:**
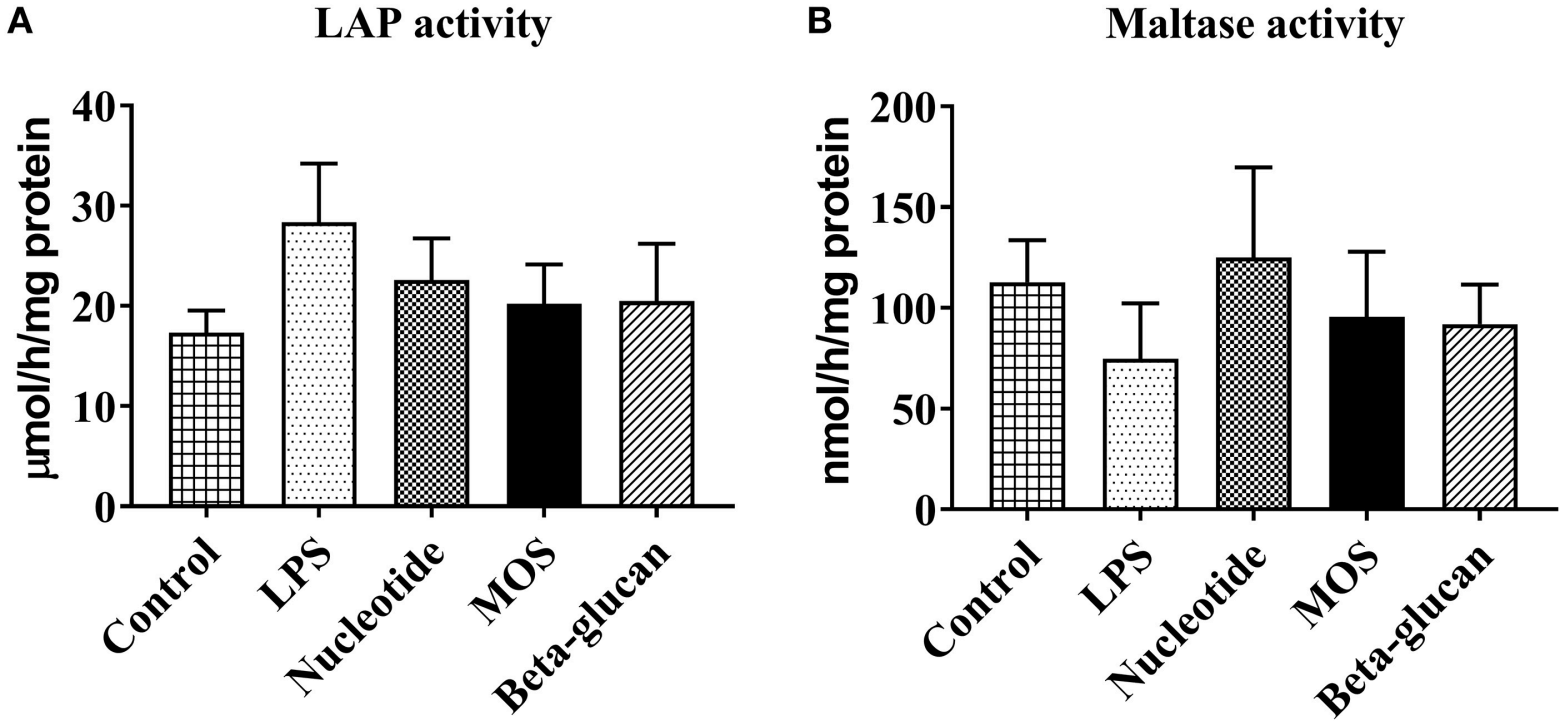
Brush border membrane leucine aminopeptidase (LAP, **A**) and maltase **(B)** activity of RTgutGC cells grown on 24-well-conventional culture plates exposed to LPS and functional ingredients. Data represent mean + SEM of two independent experiments with 3 technical well replicates each.

Brush border membrane enzyme activities (LAP and maltase) were detected in the RTgutGC cells. There were no significant effects of LPS or any of the functional ingredients on LAP (Figure 5A) or maltase (Figure 5B) activities (*P* > 0.05).

#### Gene Expression

**Figure 6:**
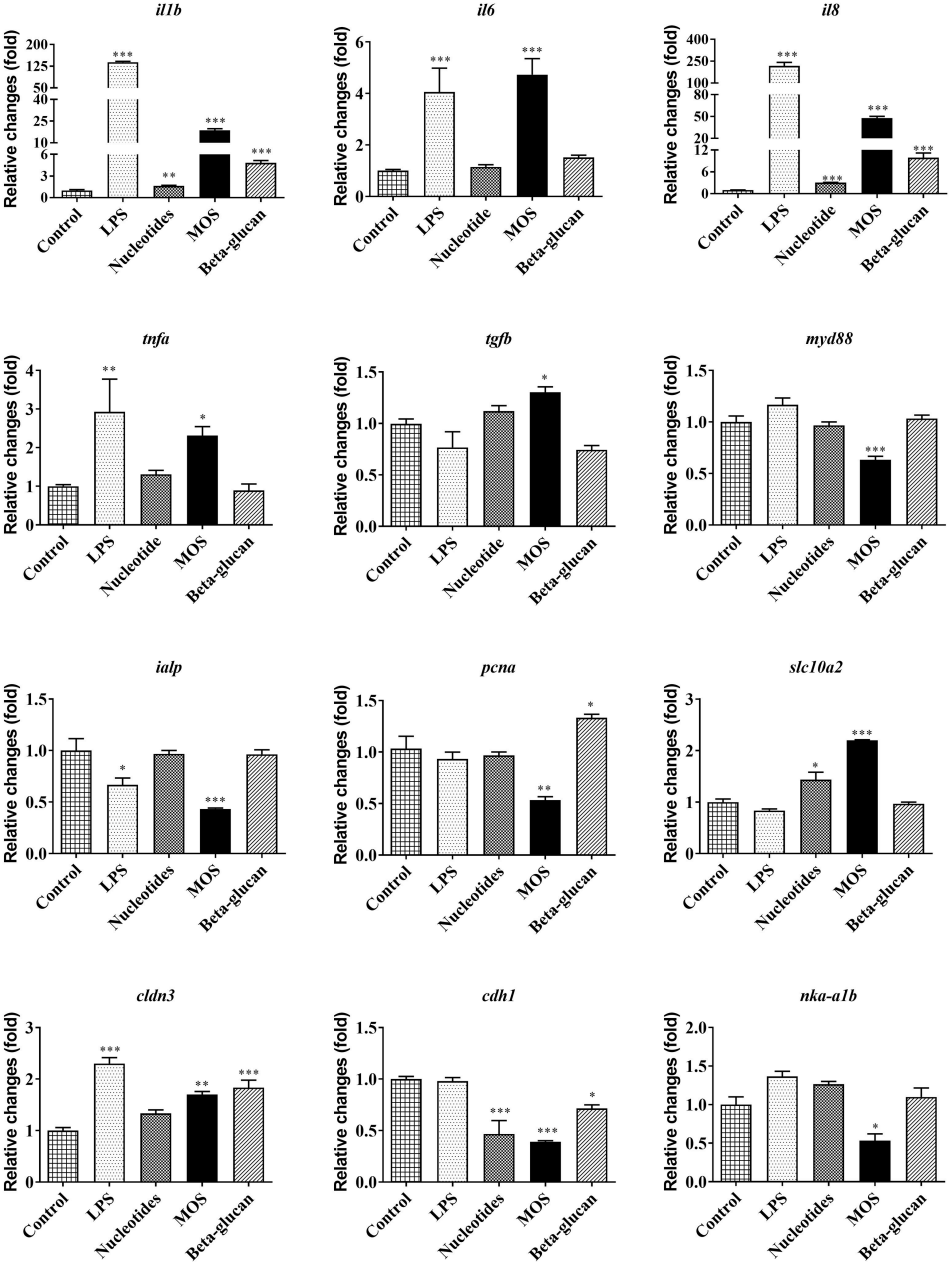
Immune, barrier, and metabolic gene expression in RTgutGC cells grown on 6-well transwell membranes exposed to LPS and functional ingredients. Data are expressed relative to control cell levels and represent mean + SEM of two independent experiments with 3 technical insert replicates each. Asterisks denote treatment groups statistically different to the control (^*^P < 0.05, ^**^P < 0.01, ^***^P < 0.001).

LPS exposure resulted in markedly increased mRNA levels of several pro-inflammatory cytokines, including interleukin 1β (*il1b*), interleukin 6 (*il6*), interleukin 8 (*il8*), and tumor necrosis factor alpha (*tnfa*). Furthermore, LPS up-regulated the expression of the tight junction gene Claudin 3 (*cldn3, P* < 0.001), but suppressed the intestinal alkaline phosphatase (*ialp)* expression (Figure 6, *P* < 0.05).

Pro-inflammatory cytokine genes (*il1b* and *il8*) were significantly increased after exposing cells to functional ingredients, especially MOS (*P* < 0.01). MOS also produced a significant up-regulation of transforming growth factor beta (*tgfb*) following 6 h of exposure (*P* < 0.05) while expression of myeloid differentiation factor 88 (*myd88*) and proliferating cell nuclear antigen (*pcna*) were significantly decreased (Figure 6, *P* < 0.01).

Compared to control, MOS and beta-glucans up-regulated the expression of *cldn3* (*P* < 0.01) while the expression of *ialp* and Na/K-ATPase (*nka*α*1b*) decreased significantly following exposure to MOS (Figure 6, *P* < 0.05). There was also a significant decrease in the expression of E-cadherin (*cdh1*) after exposure to the different functional ingredients (*P* < 0.05). Gene expression levels of the bile acid transporter solute carrier family 10 member 2 (*slc10a2*) in RTgutGC cells increased significantly after exposure to nucleotides and MOS (*P* < 0.05).

In general, immune genes were expressed at comparable relative basal levels in RTgutGC cells as in rainbow trout distal intestinal tissue, whereas most genes related to barrier function and metabolism showed lower relative expression (Supplementary Table 3). Overall, nucleotides produced little or no effect on analytical endpoints related to barrier function and gene expression. In order to reduce costs, we therefore chose to omit nucleotide exposures in the additional analyses outlined below.

#### Cell Proliferation

**Figure 7:**
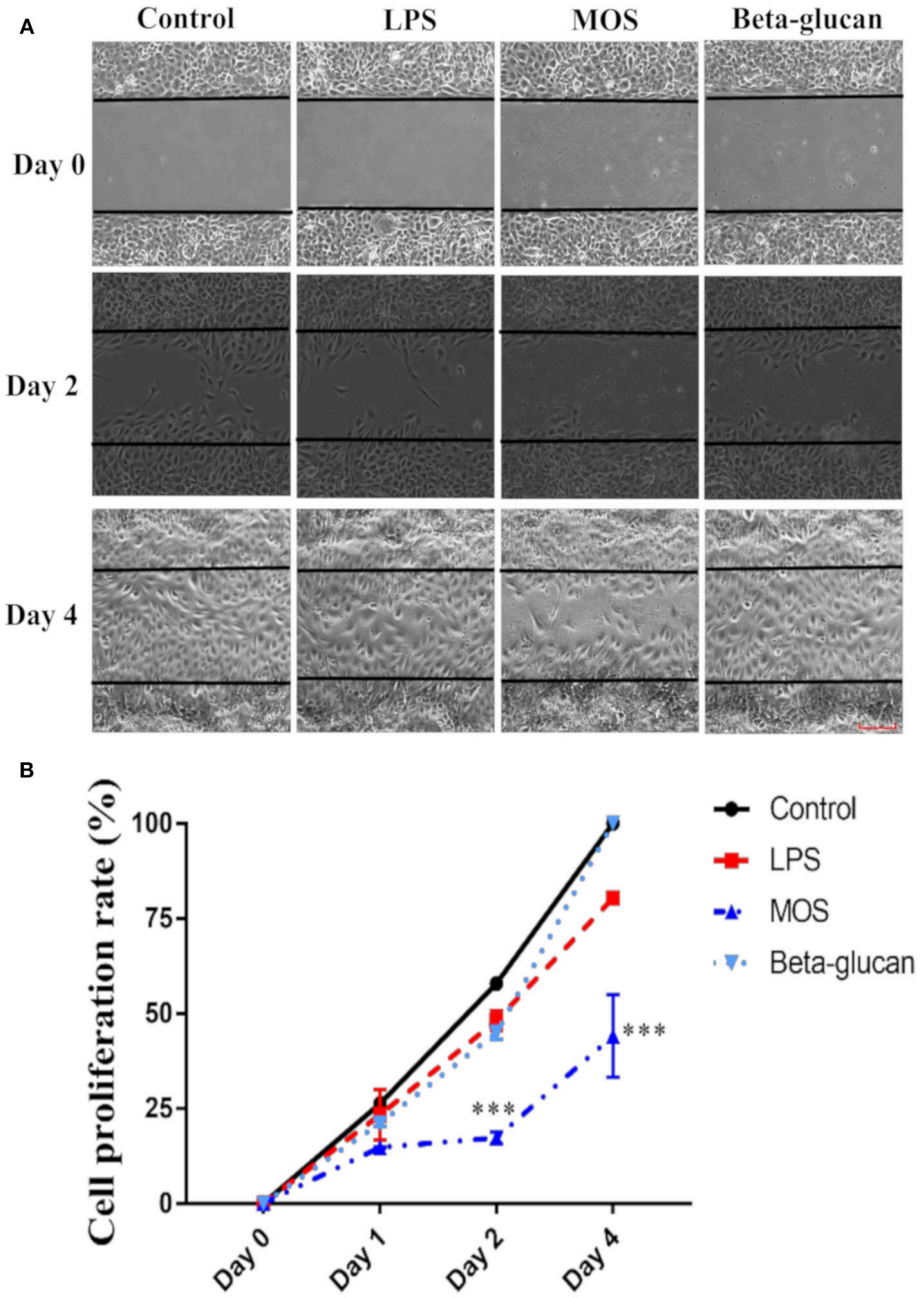
Cell proliferation assay of RTgutGC cells. **(A)** Cells treated with LPS, MOS and beta-glucan at day 0, 2, and 4 after removal of the gap insert. Representative images of three independent experiments are shown. **(B)** Quantification of cell proliferation rate at different time points during treatment. Data represent mean ± SEM of three independent experiments with 3 technical well-replicates each. Asterisks denote treatment groups statistically significant different to the control at the same time point (^***^P < 0.001). Scale bar = 100 μm.

In control cells, the gap area of the culture wells was fully closed by day 4 (Figure 7). When treated with LPS or beta-glucans, cells were able to close the gap in a similar pace as in the control cells. In contrast, MOS treatment reduced the cell proliferation and consequently the gap closure rate to < 50% at day 4 as shown in Figure 7.

#### ROS Generation

**Figure 8:**
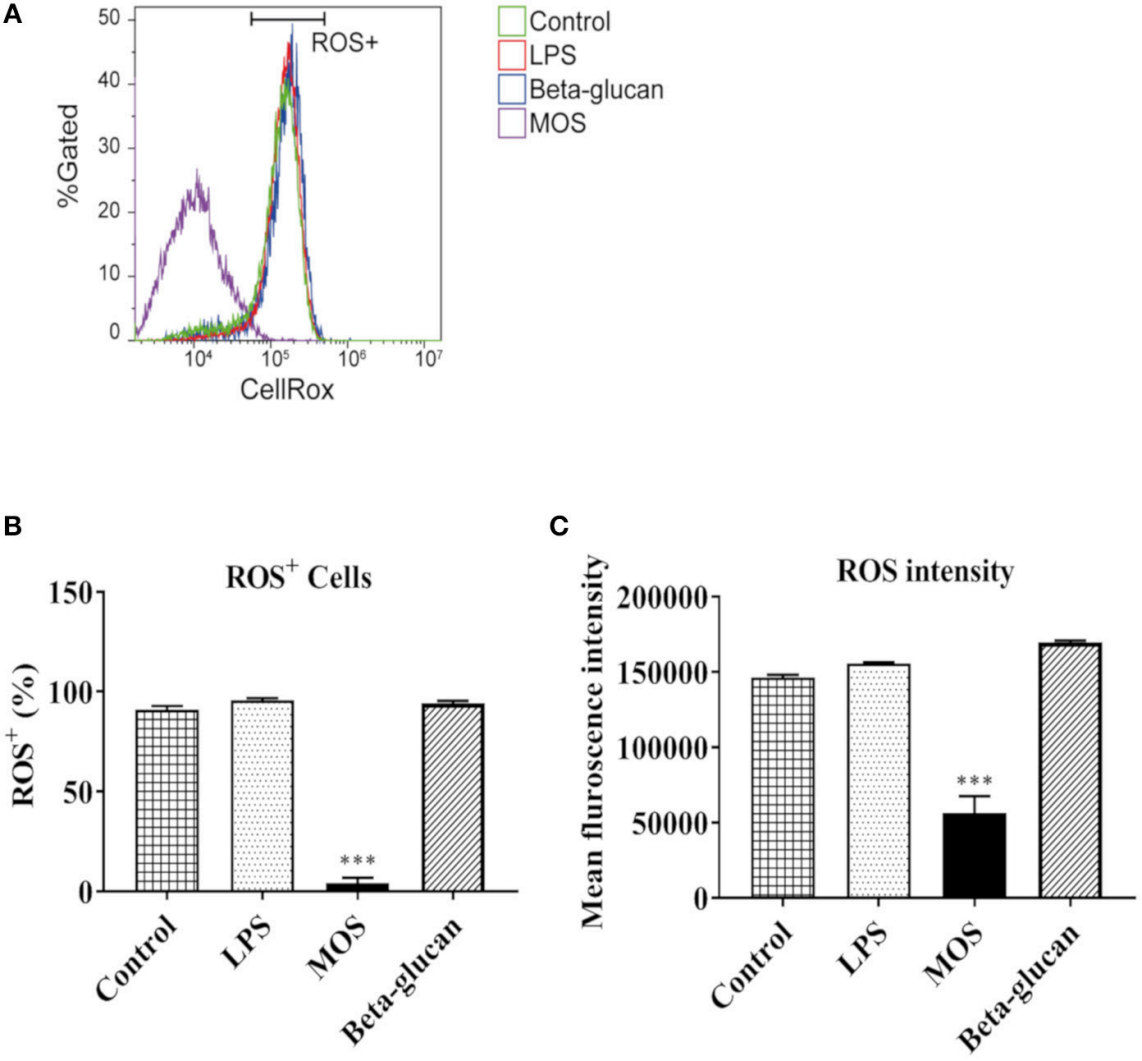
Flow cytometry analysis of reactive oxygen species (ROS) production in RTgutGC cells grown on 6-well conventional plates and exposed to LPS, MOS and beta-glucan for 6 h. **(A)** Representative histograms of ROS dye fluorescence intensity. X axis shows the CellRox fluorescence signal in cells. Y axis shows the percentage of total live cell population. Horizontal bar indicate ROS positive (ROS^+^) cells. **(B)** Statistical analysis of ROS^+^ cell number under different treatments **(C)** Mean Fluorescent Intensities of ROS dye in cells under different treatments. Data represent mean + SEM of two independent experiments with 3 technical well-replicates each. Asterisks denote treatment groups statistically significant different to the control (^***^P < 0.001).

As shown in Figures 8A–C, viable cell numbers were not affected by treatments, while MOS diminished ROS positive cells markedly (96% decreased, *P* < 0.001). Moreover, mean fluorescence intensity of ROS in cells were significantly smaller than in other groups (*P* < 0.001).

#### F-actin Content and E-cadherin, Aquaporin 8, and Hsp70 Protein Expression

**Figure 9:**
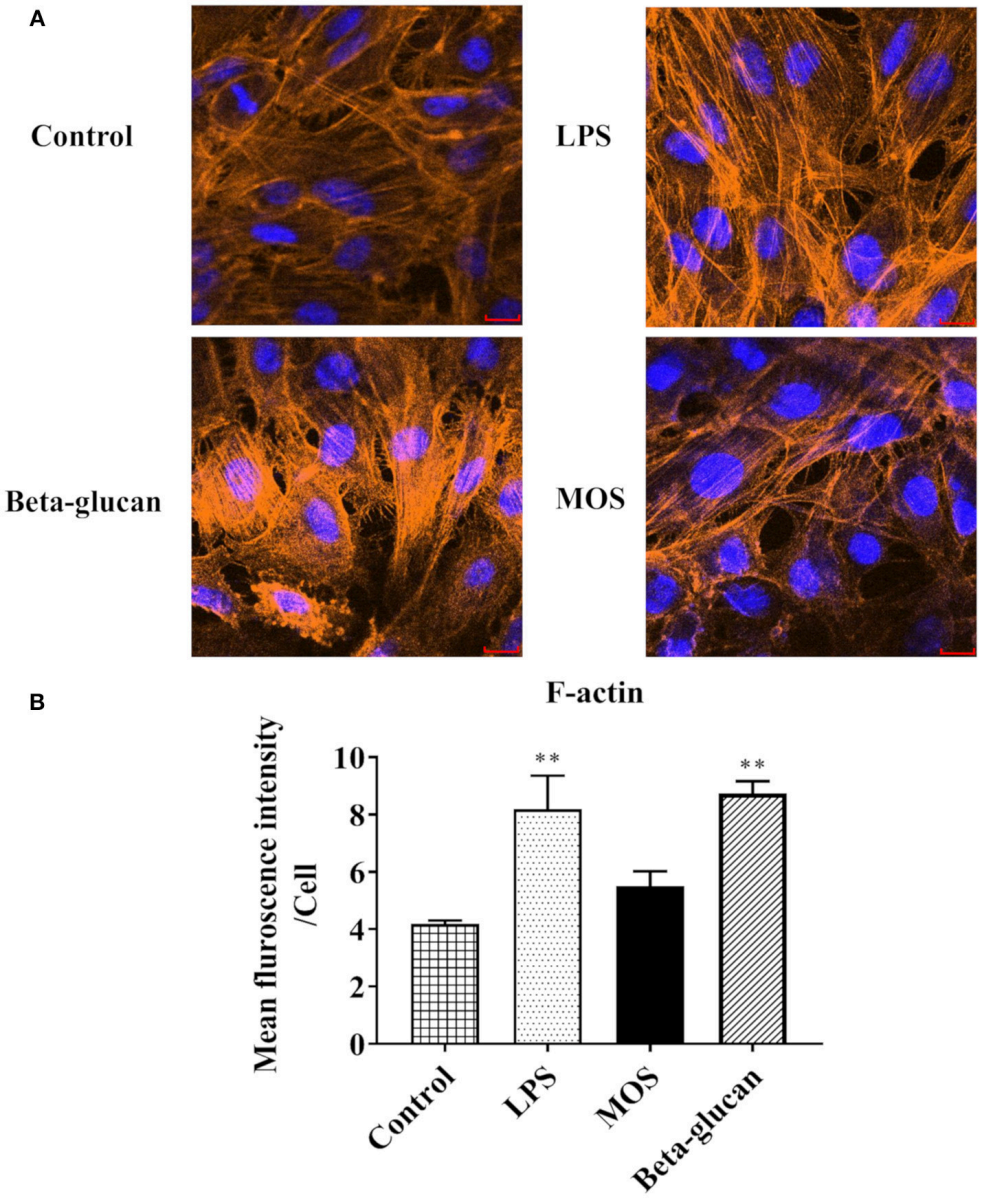
RTgutGC cell morphology after treatments with LPS, MOS, and beta-glucan. **(A)** Staining of cell nuclei (blue) and skeleton (orange) after 6 h exposure to stimulants. **(B)** Mean Fluorescent Intensities (MFI) of RTgutGC skeleton (F-actin). Data represent mean + SEM of three independent experiments, with 3 technical replicates each. Asterisks denote treatment groups statistically significant different to the control (^**^P < 0.01). Scale bar = 100 μm.

**Figure 10:**
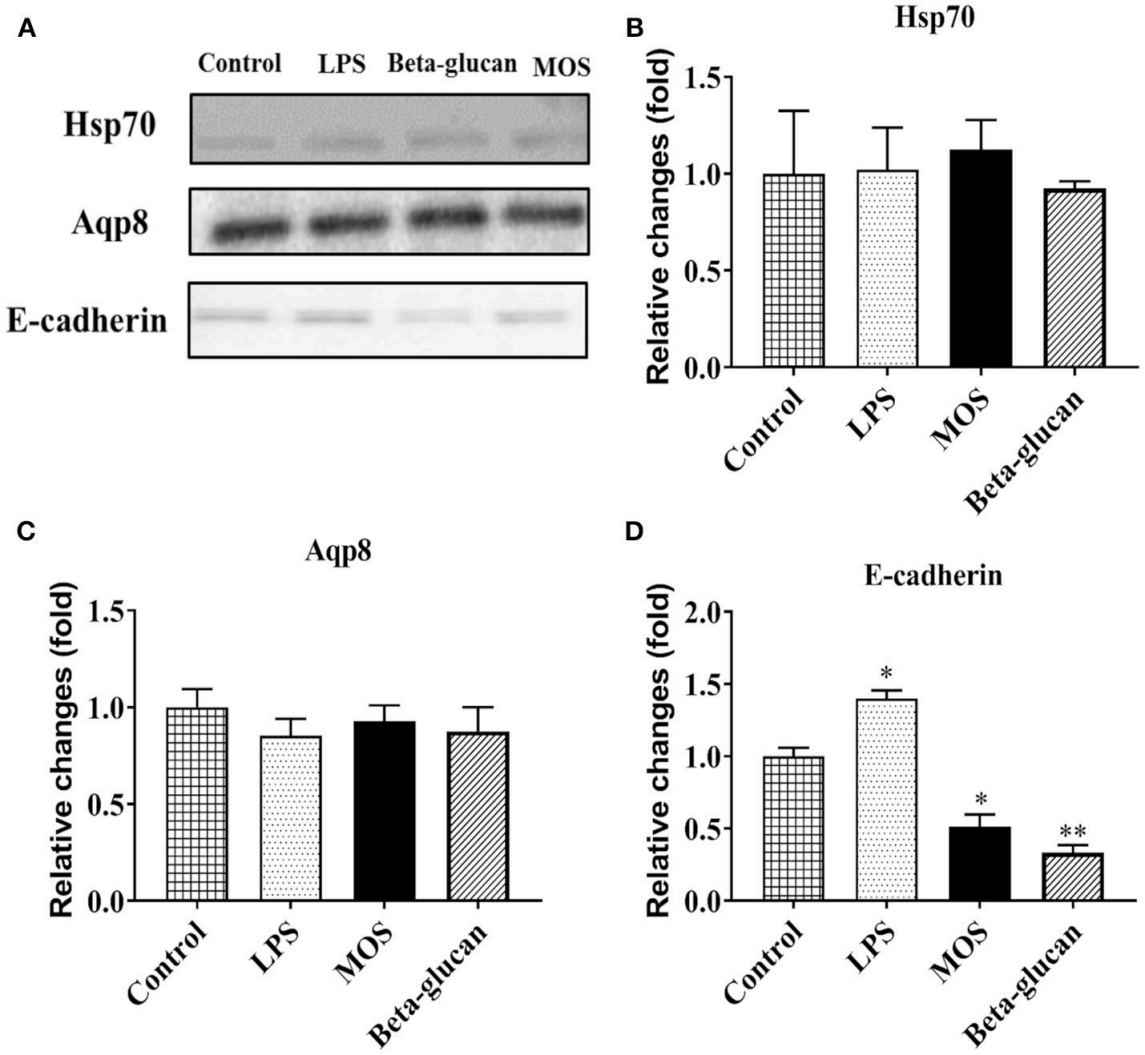
**(A)** Representative Western blot pictures for target proteins. Statistical analysis of protein expression levels of heat shock protein 70 (hsp70, **B**), aquaporin 8 (Aqp8, **C**), and e-cadherin **(D)**. Data represent mean + SEM from two or three independent experiments with 3 technical replicates each. Asterisks denote treatment groups statistically significant different to the control (^*^P < 0.05, ^**^P < 0.01). Full scans of the entire original blots are shown in Supplementary Figure 2.

As shown in Figures 9A,B, intracellular F-actin contents were significantly increased in LPS and beta-glucan groups (*P* < 0.01), while MOS treated cells remained at control levels. Western blot analyses demonstrated that expressions levels of Aqp8 and Hsp70 were not influenced by any of the treatments. E-cadherin expression was increased in cells treated with LPS, but decreased in beta-glucan and MOS treated groups (*P* < 0.01) (Figure 10).

## Discussion

Well-characterized *in vitro* model systems offer many benefits for screening purposes, given their simplicity and relative inexpensiveness compared to experiments using live animals. They could also serve as essential tools to increase the knowledge of cellular and molecular mechanisms underlying effects observed in animal trials. In the present work, we have continued the ongoing characterization of the first established intestinal epithelial cell line from fish, RTgutGC (18) and evaluated its suitability as an *in vitro* model for studies of effects of LPS and functional feed ingredients.

### Functional Characterization of RTgutGC Cells

Based on previously established RTgutGC cell features (18–23, 33), we first confirmed the viability and barrier function of the RTgutGC cells when grown on transwell membranes. Barrier formation was assessed by TEER measurements and fluorescent albumin translocation from the apical to basolateral cell chamber. TEER levels in the present study were comparable to those reported previously (19, 20). We also observed a strong and time-dependent increase in basolateral fluorescence of albumin in wells without cells, whereas low and stable values were observed for wells with cells. Thus, our observations confirmed earlier reports (20, 33) demonstrating that RTgutGC cells grown on permeable inserts strongly attenuate fluorescent model molecules' translocation from apical to basolateral chamber. RTgutGC barrier function was further supported by related gene and protein expression (*cldn3, cdh1*, Claudin 3) as previously demonstrated (19, 20, 22). We also confirmed the findings by Minghetti and co-workers (20) by demonstrating the viability of the RTgutGC cells when exposed to a buffer designed to mimic the intestinal lumen (25), i.e., mucosal saline. Another indication that the RTgutGC cells function as enterocytes is the presence of brush border membrane enzymatic activity. Previous studies have demonstrated that RTgutGC cells possess alkaline phosphatase activity (18). In the current work, we continued to explore RTgutGC brush border features by measuring activity levels of two important brush border digestive enzymes, i.e., LAP and maltase. Activity of both these enzymes were detected in the RTgutGC cells. Higher LAP activity, but very low maltase activity, were found compared to the results of *in vivo* tests (34). Altogether, the current re-establishment of key barrier and brush border features demonstrates the robustness of the RTgutGC transwell system and shows that RTgutGC cells develop certain intestinal functions similar to the *in vivo* situation.

### Effects of LPS Exposure on RTgutGC

We continued to explore RTgutGC cell immune function by detailed exposures to a prototype PAMP, i.e., LPS. LPS showing no effect on cell viability at concentrations up to 100 μg/mL is in line with previous reports suggesting that fish cells without TLR4/CD14 signaling system may be less responsive to LPS compared to mammalian cells (18, 35, 36). The LPS used in the present study was derived from *E. coli*, and it is possible that LPS isolated from a fish pathogen could be more potent in RTgutGC cells. Anyhow, the final working concentration (50 μg/mL LPS) was clearly sufficient to induce immune-related gene expression responses and influence cell proliferation and F-actin contents of RTgutGC cells in this study.

The epithelial cells of the intestinal tract are in direct contact with the external environment of the gut lumen and must be prepared to mount an immune response against antigens and infections agents of dietary origin. It is well-known that intestinal epithelial cells of teleost fish produce several innate immune defense factors, and they can over-express pro-inflammatory cytokines following a bacterial infection (23). In the present study, LPS produced markedly elevated levels of pro-inflammatory cytokine gene expression (*il1b, il6, il8*, and *tnfa*). The data point toward RTgutGC immunocompetence, and demonstrate that RTgutGC cells possess the ability and transcriptional apparatus to mount an innate immune response against LPS, a common model PAMP. There are, to our knowledge, no published studies on spatial immune gene expression patterns along the rainbow trout intestinal tract. Given that the RTgutGC cell line was initially isolated from the distal intestine (18), which is believed to be a specific intestinal region for certain mucosal immune functions (29), it is interesting to note that RTgutGC relative immune gene expression were found at comparable levels as in the distal intestine of rainbow trout (Supplementary Table 3). Induced immune transcriptional responses to pathogen infection have previously been observed in human intestinal epithelial cells (37). In fish, similar effects of LPS on innate immune related gene expression have been observed also in head kidney leukocytes of rainbow trout (9). Moreover, LPS has been reported to up-regulate *tnfa* gene expression in RTgutGC cells grown on conventional culture plates (18). Intestinal alkaline phosphatase (Ialp) is an important apical brush border enzyme, which has been found to lower the expression of pro-inflammatory cytokines by inhibiting the activation and translocation of their master transcription factor NF-κB (38). LPS is a reported substrate for Ialp (38), and in the current work, LPS suppressed *ialp* expression. This response could reflect the interplay between LPS, Ialp and pro-inflammatory cytokine signaling.

### Effects of Functional Feed Ingredients on RTgutGC

Our strategy for determining the final exposure concentrations of the functional ingredients was based on measurements of cell viability. When applied at high concentration, all functional ingredients significantly reduced cell viability in RTgutGC cells. We chose our final exposure concentrations at levels that maintained 80% cell viability as compared to control cells, with the underlying assumption that these cells were in a healthy state and could exert true physiological responses to the functional ingredients. It should be noted that the cell viability assays were performed with cells grown on conventional plates, and we therefore assume similar responses to the stimulants in cell grown on membrane inserts.

In two-compartment epithelial cell *in vitro* systems, increased TEER levels are interpreted as an increase in epithelial barrier tightness. In the present study, beta-glucans increased TEER values, whereas no significant effects were observed for nucleotides or MOS exposure. In contrast, MOS treatment increased albumin translocation across the RTgutGC monolayer, indicative of a reduced barrier function that could be attributed to alterations in both transcellular and paracellular routes. The relative proportion of trans- and paracellular translocation of albumin remains unknown, and should be explored in future studies, for example by detailed studies of albumin uptake kinetics into RTgutGC cells grown on permeable supports. Of note, we demonstrated that albumin was indeed taken up by the RTgutGC cells when grown on conventional supports, whereas no uptake of the larger molecule zymosan was detected. For junction barrier related gene expression, all functional ingredients suppressed *cdh1* levels and all ingredients except nucleotides increased *cldn3*. The suppressed *cdh1* levels in cells treated with MOS and beta-glucan were also mirrored by decreases at the protein expression level. In Caco-2 cells, decreases in *cldn3* mRNA levels were observed in concert with increase in paracellular permeability and a reduction in TEER (39). Similarly, the observed decreases in adherence junction-related *cdh1* expression would be expected to loosen the junction barrier and increase paracellular permeability. *In vivo*, MOS supplementation to fish has in several independent studies been found to improve microvilli integrity in terms of microvilli density (12) and length (12, 40, 41). In European seabass, MOS treatment enlarged intestinal fold height and reduced gut bacterial translocation, demonstrative of MOS effects on epithelial barrier function (42). Furthermore, beneficial physiological effects on epithelial cells of fish fed MOS could be a result of increasing mucus secretion (43), viscoelasticity of the mucus (44) or induced tight junction closure (ZO-1, occluding or E-cadherin) (45). To our knowledge, there are no published studies of effects of MOS on gut epithelial barrier or tight junction function in rainbow trout. The findings of TEER, albumin translocation, and junction barrier related gene and protein expression in the present study may point to how MOS can act as homeostatic balancer of barrier function *in vivo* and *in vitro* (46).

RTgutGC cell proliferation was assessed by a previously established cell proliferation assay (22). RTgutGC cells had the ability to close the cell free gap in 4 days in this study, which was faster than in a previous report, possibly due to different culture conditions (22). During the 4-day period, MOS strongly reduced the cell proliferation speed compared to control. In addition, MOS significantly suppressed ROS production compared with control cells. ROS plays important roles in homeostasis and cell signaling, and ROS levels typically increase during periods of environmental stress and may cause significant damage to cell structures (47). Whether the MOS-induced decrease in RTgutGC proliferation ability could be a result of reduction in stress fibers and suppressed ROS production as previously reported (48, 49) warrants further investigation. Pcna plays an important role in cell proliferation (50). MOS also down-regulated *pcna* gene expression in the present study, confirming the cell proliferation assay results indicating that MOS inhibited cell proliferation (50).

Functional ingredients are expected to exert immune-modulatory effects in the intestine by regulating the expression of cytokines (2, 7, 10, 51). Among the functional ingredients evaluated in the present work, MOS seemed to be the most potent modulator of RTgutGC immune responses. Specifically, MOS treatment induced levels of pro-inflammatory (*il1b, il6, il8*, and *tnfa*) and *tgfb* cytokine transcripts, but suppressed *myd88* expression. In particular, the alterations of pro-inflammatory cytokine gene expression and the suppression of *ialp* mirrored the effect of LPS. *In vivo*, dietary MOS in European sea bass can provide protection against *Vibrio alginolyticus* infection (52) and counteract the side effects of soybean meal oil by increasing the mucus cell density and area in the distal intestine and regulating GALT-related genes (i.e. *il6, il10*, and *tgfb*) (46). MOS supplementation to rainbow trout was also found to improve lysozyme concentration, classical pathway of complement (APCA and CPCA) (53), microvilli structure and absorptive surface area (12). Whether the immune-modulatory effects induced by MOS in the present study having any relation to the increase in epithelial permeability is a question that clearly warrants attention in future studies. Possibly, the increased permeability could lead to an increased antigen influx that would trigger mucosal immune responses, including modulation of cytokine expression.

Beta-glucan is one of the potent and promising immunostimulants in aquaculture which could be beneficial for growth, disease resistance and immune response of a range of fish species including rainbow trout (54–56). *In vitro*, beta-glucans were found to have positive effects on neutrophil degranulation of fathead minnows (57) and respiratory burst activity of Atlantic salmon (58). In the present study, beta-glucan treatment also produced increased mRNA levels of pro-inflammatory cytokine genes (*il1b* and *il8*). This observation is in agreement with previous studies demonstrating that beta-glucans up-regulated pro-inflammatory cytokine expression in head kidney cells of rainbow trout (9) and increased *il1b* expression in Atlantic cod after challenged with *Vibrio anguillarum* (10). A previous report also found that *il1b* production was induced by cathelicidin-2 variants and *il1b* expression upregulation was elicited by a synergic effect of zymosan and cathelicidin-2 variants in RTgutGC cells (23). Beta-glucan lowered transactivation of NF-κB to stimulate immune response was also found in Caco-2 cells (15). Whether the expression of *il1b* and *il8* is affected by the cathelicidin-2 variants or the activation of NF-κB in RTgutGC still needs to be explored in future studies. *In vivo*, the expression of *il8* was not affected significantly in the distal intestine of Atlantic cod fed beta-glucans (10), which is different from our findings. Available literature suggests that beta-glucans may regulate inflammatory effects in an inconsistent pattern, possibly depending on the differences of composition, dosage, quality, route, and exposure time (11, 23, 54, 59). Nucleotides also produced elevated levels of *il1b* and *il8*, but the degree of response was minor compared to the other functional ingredients evaluated in the current study. Previous *in vivo* tests have found that dietary nucleotides might improve growth, disease resistance against *S. iniae* and pancreatic necrosis, serum alternative complement activity, serum lysozyme activity and crowding stress of rainbow trout (60–62) and influence macrophage activity, respiratory burst activity and expression of *il1b, il8*, and *tnfa* in turbot (63). However, the mechanism of growth and immune promotion by nucleotides still need to be identified *in vitro* or *in vivo* tests.

## Conclusion

An increasing body of literature demonstrates that functional feed ingredients can support intestinal health and reduce disease susceptibility via multiple mechanisms, including direct effects on a variety of intestinal functions, e.g., barrier function, nutrient transport and immune responses (7, 23, 64–66). In fish, knowledge about basic mechanisms of functional ingredients and their interactions with the intestinal tissue is weak and fragmentary. The present study has provided new information on how functional ingredients commonly applied in aquafeeds can affect intestinal epithelial function in fish. Additionally, our study demonstrates the suitability of the RTgutGC transwell system as an alternative to fish feeding experiments for prediction of health effects of functional feeds.

## Author Contributions

JW, PL, AG, YY, ÅK, and TK: experiment design; JW, PL, AG, LL, YY, and TK: analyses; KS, LM, MØ, ÅK, and TK: supervision; JW and TK: writing, original draft; JW, PL, AG, LL, YY, KS, LM, MØ, ÅK, and TK: writing, review, and editing.

### Conflict of Interest Statement

The authors declare that the research was conducted in the absence of any commercial or financial relationships that could be construed as a potential conflict of interest.
