# Supplementary material for: Rainbow Trout (Oncorhynchus Mykiss) Intestinal Epithelial Cells as a Model for Studying Gut Immune Function and Effects of Functional Feed Ingredients
Source: Front Immunol. 2019 Feb 6;10:152. doi: 10.3389/fimmu.2019.00152 (PMC6374633; doi:10.3389/fimmu.2019.00152)
Supplement: Supplementary file 1 [file Data_Sheet_1.docx]

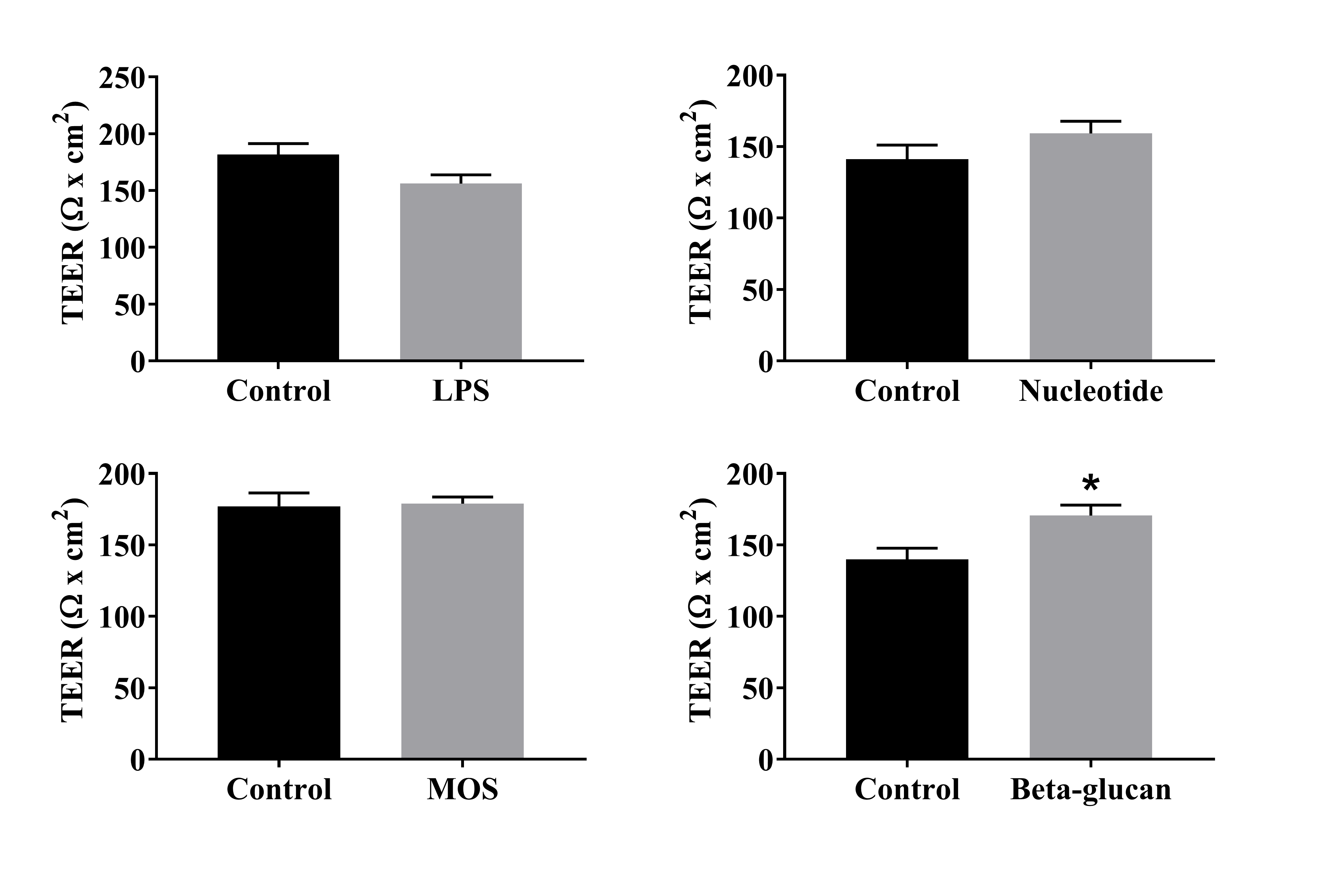


**Supplementary Figure 1:** TEER levels of RTgutGC cultures exposed to LPS and functional ingredients in 6-well transwell membrane inserts for 6 hours. Data are expressed as Ω × cm^2^ and represent mean + SEM of two independent experiments with 3 technical insert replicates each. Asterisks denote treatment groups statistically different to the control (* *P*<0.05).
