# Supplementary material for: Rainbow Trout (Oncorhynchus Mykiss) Intestinal Epithelial Cells as a Model for Studying Gut Immune Function and Effects of Functional Feed Ingredients
Source: Front Immunol. 2019 Feb 6;10:152. doi: 10.3389/fimmu.2019.00152 (PMC6374633; doi:10.3389/fimmu.2019.00152)
Supplement: Supplementary file 2 [file Data_Sheet_2.docx]

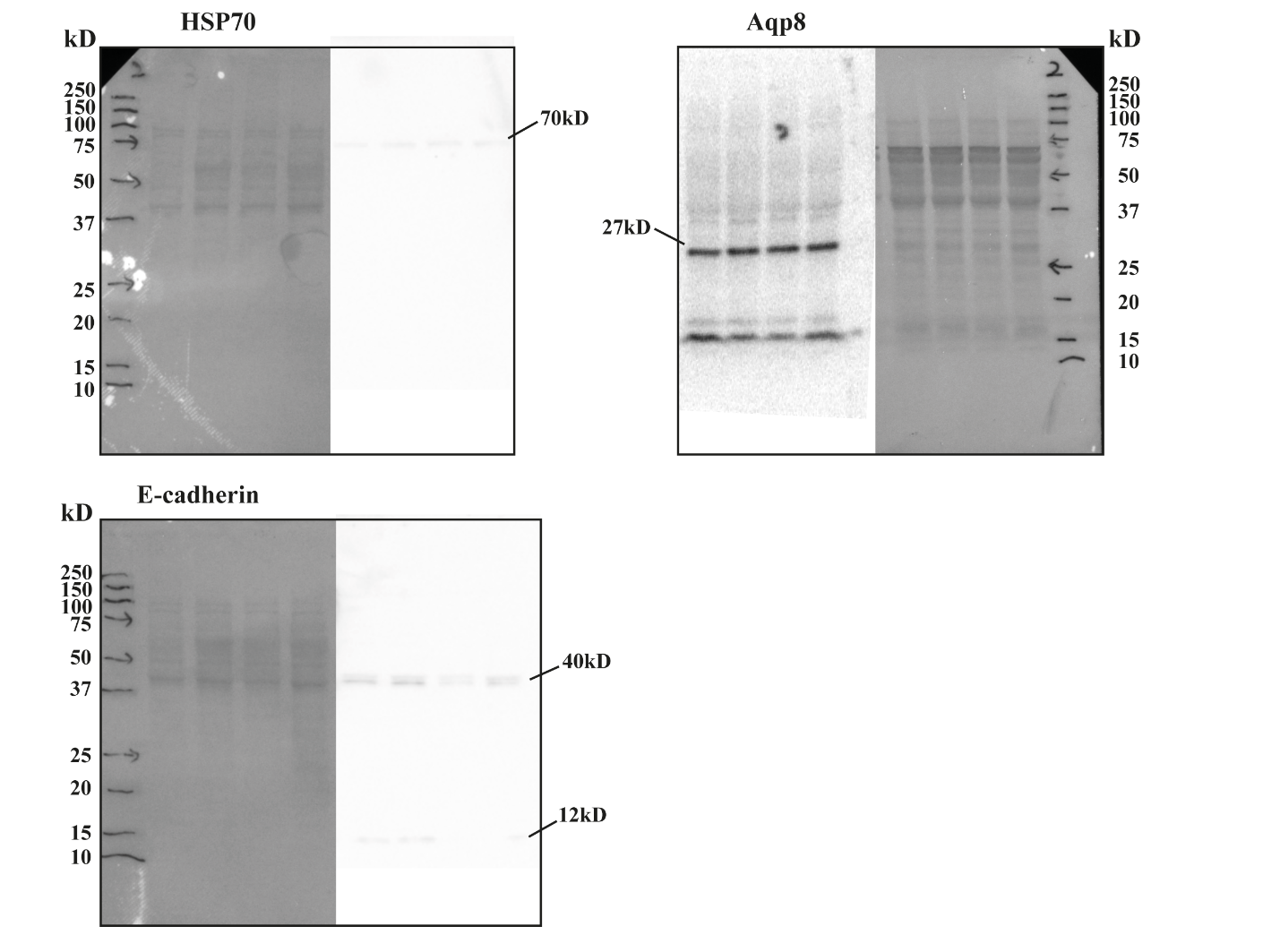


**Supplementary Figure 2:** Original Western blot scannings showing the expression of Heat shock protein 70 (Hsp70), Aquaporin 8 (Aqp8) and E-cadherin. Equal amount of proteins (20µg for Aqp8, 10 µg for Hsp70 and E-cadherin) extracted from RTgutGC cells after different treatments (Control, LPS, Beta-glucan and MOS) were loaded for each blot. For Hsp70 and E-cadherin, the loading sequence (left to right) is: protein ladder, control, LPS, Beta-glucan, MOS. For Aqp8, the loading sequence (left to right) is: MOS, Beta-glucan, LPS, control. The ponceau staining images were used as indication of total protein loading amounts, and are displayed to the left (Hsp70, E-cadherin) or right (Aqp8) of the antibody blots. Samples were resolved on a 12% gel and Western blotting was performed using respective antibodies. The positions of Hsp70 (70kD), Aqp8 (27kD) and E-cadherin (12kDa, 40kDa) are indicated.
