# Supplementary material for: Rainbow Trout (Oncorhynchus Mykiss) Intestinal Epithelial Cells as a Model for Studying Gut Immune Function and Effects of Functional Feed Ingredients
Source: Front Immunol. 2019 Feb 6;10:152. doi: 10.3389/fimmu.2019.00152 (PMC6374633; doi:10.3389/fimmu.2019.00152)
Supplement: Supplementary file 3 [file Data_Sheet_3.docx]

| **Supplementary Table 1 \| Preparation of mucosal saline** | | | | | |
| --- | --- | --- | --- | --- | --- |
| **Substance** | **mM** | **MW** | **gr./L** | **Water added** | **Supplier (product number)** |
| NaCl | 69 | 58.44 | 4.03 | To a final volume of: 1000 mL | Sigma (S5886-500G) |
| KCl | 5 | 74.55 | 0.373 |  | Sigma (P5405-250G) |
| MgSO_4_ | 77.5 | 120.37 | 9.329 |  | Sigma (M2643-500G) |
| MgCl_2_ | 22.5 | 95.22 | 2.14 |  | Sigma (M4880-100G) |
| CaCl_2_ | 5 | 110.99 | 0.555 |  | Sigma (C5670-100G) |

Note: Buffer preparation was made and autoclaved prior to usage according to the description of Genz et al. (2011). The pH of the buffer was 7.2.

Genz, J., Esbaugh, A. J. and Grosell, M. (2011) 'Intestinal transport following transfer to increased salinity in an anadromous fish (Oncorhynchus mykiss)', *Comparative Biochemistry and Physiology a-Molecular & Integrative Physiology,* 159(2), 150-158.
