# Supplementary material for: Rainbow Trout (Oncorhynchus Mykiss) Intestinal Epithelial Cells as a Model for Studying Gut Immune Function and Effects of Functional Feed Ingredients
Source: Front Immunol. 2019 Feb 6;10:152. doi: 10.3389/fimmu.2019.00152 (PMC6374633; doi:10.3389/fimmu.2019.00152)
Supplement: Supplementary file 4 [file Data_Sheet_4.docx]

| **Supplementary Table 2 \| Concentrations of stimulant solutions** | | | | | | | |
| --- | --- | --- | --- | --- | --- | --- | --- |
| **Stimulants** | **LPS^a^ concentration**  **(µg/mL)** | **Nucleotide^b^**  **concentration**  **(µg/mL)** | | **MOS^b^ concentration**  **(µg/mL)** | | **Beta-glucan^b^ concentration**  **(µg/mL)** | |
| Working solutions | 1 | 10 | 50 | 10 | 500 | 1 | 10 |
|  | 2.5 | 50 | 75 | 100 | 1000 | 10 | 20 |
|  | 10 | 100 | 100 | 500 | 2000 | 25 | 40 |
|  | 25 | 250 | 150 | 1000 | 3000 | 50 | 60 |
|  | 50 | 500 | 200 | 5000 | 4000 | 100 | 80 |
|  | 100 | 1000 | 250 | 10000 | 5000 | 250 | 100 |

^a^ Two independent experiments with identical exposure concentrations were performed

^b^ Two independent experiments with overlapping exposure concentrations were performed (left and right columns)
